# Supplementary material for: A New Approach for Determination of the Botanical Origin of Monofloral Bee Honey, Combining Mineral Content, Physicochemical Parameters, and Self-Organizing Maps
Source: Molecules. 2021 Nov 28;26(23):7219. doi: 10.3390/molecules26237219 (PMC8659082; doi:10.3390/molecules26237219)
Supplement: Supplementary file 1 [file molecules-26-07219-s001.zip › Table S1.pdf]

Table S1. The data matrix of physicochemical parameters and minerals in acacia bee honey samples (n_{2018}=2 and n_{2019}=2).

Analyte	Unit	2018		2019	
		Sample 1	Sample 2	Sample 1	Sample 2
**Col**	**mm Pfund**	11	10	2	3
**Cond**	**mS/cm**	0.580	0.575	0.123	0.115
**Diast**	**DN**	14.25	14.90	17.38	17.89
**HMF**	**mg/kg**	2.50	3.13	2.38	2.15
**Invert**	**U/kg**	81.06	79.57	82.65	81.30
**pH**	**-**	3.75	3.70	3.65	3.55
**Prol**	**mg/kg**	236.40	240.15	269.07	255.89
**Rot**	$[\alpha]_D^{20}$	-15.00	-16.25	-21.25	-21.75
**Water**	**%**	17.20	17.00	18.10	18.00
**Ag**	**µg/kg**	< LOD*			
**Al**	**mg/kg**	0.44	0.36	0.49	0.68
**As**	**µg/kg**	< LOD*			
**B**	**mg/kg**	3.1	3.7	2.8	3.9
**Ba**	**µg/kg**	29	110	22	27
**Bi**	**µg/kg**	0.194	0.055	0.086	0.078
**Ca**	**mg/kg**	28	42	25	22
**Cd**	**µg/kg**	0.190	0.140	0.136	0.132
**Co**	**µg/kg**	1.03	1.01	1.37	1.28
**Cr**	**µg/kg**	5.2	3.8	18	21
**Cs**	**µg/kg**	0.20	0.35	0.22	0.44
**Cu**	**µg/kg**	109	120	82	102
**Fe**	**mg/kg**	0.58	1.09	0.29	1.74
**Ga**	**µg/kg**	0.139	0.110	0.111	0.30
**In**	**µg/kg**	0.051	0.39	0.010	0.21
**K**	**mg/kg**	246	312	256	312
**Li**	**µg/kg**	0.98	4.7	0.80	9.5
**Mg**	**mg/kg**	9.5	15	8.8	7.4
**Mn**	**mg/kg**	0.12	0.14	0.11	0.13
**Na**	**mg/kg**	12	10	15	12
**Ni**	**µg/kg**	52	245	48	222
**P**	**mg/kg**	34	36	35	36
**Pb**	**µg/kg**	18	67	12	57
**Rb**	**µg/kg**	191	253	183	231

**S**	**mg/kg**	13	16	15	15
**Se**	**µg/kg**	< LOD*			
**Sr**	**mg/kg**	0.049	0.069	0.043	0.038
**Te**	**µg/kg**	< LOD*			
**V**	**µg/kg**	< LOD*	0.27	< LOD*	0.068
**Zn**	**mg/kg**	1.13	1.12	0.91	0.81

*LOD = 0.0001 µg/kg
