# Supplementary material for: A New Approach for Determination of the Botanical Origin of Monofloral Bee Honey, Combining Mineral Content, Physicochemical Parameters, and Self-Organizing Maps
Source: Molecules. 2021 Nov 28;26(23):7219. doi: 10.3390/molecules26237219 (PMC8659082; doi:10.3390/molecules26237219)
Supplement: Supplementary file 1 [file molecules-26-07219-s001.zip › Table S2.pdf]

Table S2. Basic statistics of physicochemical parameters and minerals in linden bee honey samples (n_{2018}=17 and n_{2019}=14).

Analyte	Unit	Min		Max		Mean		St. dev.	
		**2018**	**2019**	**2018**	**2019**	**2018**	**2019**	**2018**	**2019**
**Col**	**mm Pfund**	4	3	35	35	20.46	14.08	8.74	8.94
**Cond**	**mS/cm**	0.58	0.39	0.79	0.79	0.67	0.63	0.07	0.13
**Diast**	**DN**	10.16	11.05	21.45	24.55	16.06	17.83	3.08	3.54
**HMF**	**mg/kg**	1.80	1.94	8.38	7.93	5.72	5.21	1.85	2.14
**Invert**	**U/kg**	75.97	63.58	175.15	145.59	119.36	104.87	26.07	25.39
**pH**	**-**	3.70	3.90	4.65	4.70	4.29	4.29	0.25	0.29
**Prol**	**mg/kg**	237.71	216.38	532.22	518.72	348.40	331.94	96.67	84.02
**Rot**	$[\alpha]_D^{20}$	-20.00	-20.00	-11.50	-10.50	-14.92	-13.71	3.25	2.50
**Water**	**%**	16.70	16.00	19.80	19.60	17.89	17.90	0.91	1.08
**Ag**	**µg/kg**	< LOD*							
**Al**	**mg/kg**	0.59	0.51	1.26	1.42	0.89	0.81	0.21	0.28
**As**	**µg/kg**	< LOD*							
**B**	**mg/kg**	1.81	1.29	5.8	9.5	3.4	4.9	1.10	3.0
**Ba**	**µg/kg**	133	19	753	289	265	152	169	96
**Bi**	**µg/kg**	0.023	0.020	0.21	0.058	0.088	0.038	0.058	0.014
**Ca**	**mg/kg**	56	56	177	134	111	90	33	23
**Cd**	**µg/kg**	0.180	0.089	1.46	0.88	0.78	0.51	0.33	0.30
**Co**	**µg/kg**	0.86	0.82	74	31	9.4	6.0	21	9.5
**Cr**	**µg/kg**	4.3	4.4	14	15	7.8	7.6	2.9	4.0
**Cs**	**µg/kg**	0.62	0.72	2.3	2.9	1.28	1.75	0.57	0.76
**Cu**	**µg/kg**	120	119	333	381	203	199	66	92
**Fe**	**mg/kg**	0.82	0.77	7.2	3.6	3.1	1.75	2.1	0.84
**Ga**	**µg/kg**	0.200	0.083	0.54	0.36	0.29	0.200	0.097	0.101
**In**	**µg/kg**	0.0024	< LOD*	3.3	4.4	0.45	0.52	0.94	1.36
**K**	**mg/kg**	1277	332	2339	2089	1599	1227	351	667
**Li**	**µg/kg**	2.6	1.45	46	47	15	14	12	15
**Mg**	**mg/kg**	27	19	51	47	35	31	6.8	8.8
**Mn**	**mg/kg**	0.26	0.101	2.8	2.6	1.04	0.86	0.73	0.92
**Na**	**mg/kg**	10	8	59	20	20	12	13	3.7
**Ni**	**µg/kg**	15	16	167	75	51	40	45	17
**P**	**mg/kg**	34	38	90	78	56	50	16	14
**Pb**	**µg/kg**	21	15	2149	203	262	78	596	68
**Rb**	**µg/kg**	1003	701	2422	2356	1491	1433	380	581

**S**	**mg/kg**	17	14	37	35	26	23	6.6	6.6
**Se**	**µg/kg**	< LOD*							
**Sr**	**mg/kg**	0.189	0.082	0.33	0.28	0.25	0.173	0.052	0.073
**Te**	**µg/kg**	< LOD*							
**V**	**µg/kg**	0.42	0.193	3.1	1.54	1.41	1.07	0.75	0.48
**Zn**	**mg/kg**	0.86	0.64	25	5.3	3.5	1.52	6.9	1.44

*LOD = 0.0001 µg/kg
