# Supplementary material for: A New Approach for Determination of the Botanical Origin of Monofloral Bee Honey, Combining Mineral Content, Physicochemical Parameters, and Self-Organizing Maps
Source: Molecules. 2021 Nov 28;26(23):7219. doi: 10.3390/molecules26237219 (PMC8659082; doi:10.3390/molecules26237219)
Supplement: Supplementary file 1 [file molecules-26-07219-s001.zip › Table S3.pdf]

Table S3. Basic statistics of physicochemical parameters and minerals in sunflower bee honey samples from 2019 (n=10).

Analyte	Unit	Min	Max	Mean	St. dev.
**Col**	**mm Pfund**	4	10	7.10	2.02
**Cond**	**mS/cm**	0.26	0.42	0.35	0.06
**Diast**	**DN**	7.00	17.80	10.87	3.63
**HMF**	**mg/kg**	3.55	22.40	12.85	7.02
**Invert**	**U/kg**	57.03	97.75	80.27	14.37
**pH**	**-**	3.50	3.80	3.63	0.09
**Prol**	**mg/kg**	420.50	603.48	495.05	84.55
**Rot**	$[\alpha]_D^{20}$	-20.00	-17.50	-18.90	1.02
**Water**	**%**	17.60	18.40	18.10	0.25
**Ag**	**µg/kg**	< LOD*			
**Al**	**mg/kg**	0.48	0.91	0.69	0.172
**As**	**µg/kg**	< LOD*			
**B**	**mg/kg**	2.6	11	6.5	3.8
**Ba**	**µg/kg**	16	100	40	31
**Bi**	**µg/kg**	0.020	0.076	0.051	0.018
**Ca**	**mg/kg**	17	111	73	39
**Cd**	**µg/kg**	0.094	0.84	0.42	0.24
**Co**	**µg/kg**	0.68	4.0	1.63	1.14
**Cr**	**µg/kg**	3.3	12	8.0	3.2
**Cs**	**µg/kg**	0.27	2.2	1.13	0.68
**Cu**	**µg/kg**	100	157	119	17
**Fe**	**mg/kg**	0.58	1.34	1.00	0.27
**Ga**	**µg/kg**	0.021	0.167	0.094	0.055
**In**	**µg/kg**	< LOD*	5.1	0.52	1.61
**K**	**mg/kg**	334	1302	698	342
**Li**	**µg/kg**	0.58	7.0	2.5	2.6
**Mg**	**mg/kg**	8.1	35	23	10
**Mn**	**mg/kg**	0.17	1.93	0.54	0.72
**Na**	**mg/kg**	6.0	13	8.5	2.5
**Ni**	**µg/kg**	1.04	25	14	9.0
**P**	**mg/kg**	38	45	41	2.8
**Pb**	**µg/kg**	5.3	63	14	18
**Rb**	**µg/kg**	175	1299	930	414
**S**	**mg/kg**	18	23	20	1.61

**Se**	**µg/kg**	< LOD*			
**Sr**	**mg/kg**	0.028	0.162	0.091	0.049
**Te**	**µg/kg**	< LOD*			
**V**	**µg/kg**	< LOD*	0.68	0.195	0.23
**Zn**	**mg/kg**	0.46	1.14	0.78	0.23

*LOD = 0.0001 µg/kg
