# Supplementary material for: A New Approach for Determination of the Botanical Origin of Monofloral Bee Honey, Combining Mineral Content, Physicochemical Parameters, and Self-Organizing Maps
Source: Molecules. 2021 Nov 28;26(23):7219. doi: 10.3390/molecules26237219 (PMC8659082; doi:10.3390/molecules26237219)
Supplement: Supplementary file 1 [file molecules-26-07219-s001.zip › Table S4.pdf]

Table S4. Basic statistics of physicochemical parameters and minerals in rapeseed bee honey samples (n_{2018}=8 and n_{2019}=6).

Analyte	Unit	Min		Max		Mean		St. dev.	
		**2018**	**2019**	**2018**	**2019**	**2018**	**2019**	**2018**	**2019**
**Col**	**mm Pfund**	1	2	12	17	3.71	3.60	3.73	6.11
**Cond**	**mS/cm**	0.11	0.12	0.15	0.20	0.12	0.15	0.01	0.03
**Diast**	**DN**	18.90	19.92	26.78	29.86	22.93	23.95	3.54	3.76
**HMF**	**mg/kg**	1.05	3.29	7.18	6.58	4.51	5.44	2.38	1.32
**Invert**	**U/kg**	46.09	64.85	149.88	82.17	96.66	77.12	37.41	7.16
**pH**	**-**	3.50	3.50	4.00	3.80	3.68	3.68	0.17	0.13
**Prol**	**mg/kg**	156.39	153.76	333.70	207.64	239.58	180.57	67.19	22.49
**Rot**	$[\alpha]_D^{20}$	-27.50	-25.00	-17.50	-17.50	-21.36	-20.40	3.93	2.97
**Water**	**%**	15.80	16.80	19.40	18.40	17.39	17.64	1.38	0.64
**Ag**	**µg/kg**	< LOD*							
**Al**	**mg/kg**	0.38	0.33	0.97	0.68	0.68	0.57	0.27	0.164
**As**	**µg/kg**	< LOD*							
**B**	**mg/kg**	3.5	4.3	6.1	7.8	5.2	5.8	0.94	1.68
**Ba**	**µg/kg**	89	83	121	107	103	94	11	10.0
**Bi**	**µg/kg**	0.040	0.020	0.113	0.089	0.085	0.057	0.029	0.029
**Ca**	**mg/kg**	32	31	47	72	41	48	6.0	18
**Cd**	**µg/kg**	0.20	0.146	1.07	0.70	0.49	0.31	0.30	0.27
**Co**	**µg/kg**	0.87	1.18	3.8	10	2.1	4.0	1.23	4.2
**Cr**	**µg/kg**	2.4	2.8	8.3	8.1	5.5	5.7	2.3	2.2
**Cs**	**µg/kg**	0.37	0.33	0.47	0.46	0.41	0.39	0.045	0.056
**Cu**	**µg/kg**	67	68	100	111	87	93	12	21
**Fe**	**mg/kg**	0.43	0.60	1.46	3.0	1.17	1.68	0.40	0.97
**Ga**	**µg/kg**	0.095	0.112	0.27	0.173	0.198	0.156	0.065	0.029
**In**	**µg/kg**	0.00014	0.027	0.075	0.25	0.036	0.099	0.031	0.104
**K**	**mg/kg**	153	169	200	214	174	190	18	21
**Li**	**µg/kg**	1.68	2.3	3.3	4.2	2.6	3.4	0.60	0.90
**Mg**	**mg/kg**	10	11	13	16	12	14	1.13	2.3
**Mn**	**mg/kg**	0.070	0.071	0.181	0.202	0.141	0.133	0.045	0.054
**Na**	**mg/kg**	5.2	4.4	16	11	12	8.2	4.7	3.1
**Ni**	**µg/kg**	30	28	118	251	61	89	36	108
**P**	**mg/kg**	25	31	36	38	32	34	4.2	2.9
**Pb**	**µg/kg**	99	21	179	205	117	143	31	83

**Rb**	**µg/kg**	172	171	251	270	210	229	31	44
**S**	**mg/kg**	8.5	9.9	14	16	10	12	2.5	2.7
**Se**	**µg/kg**	< LOD*							
**Sr**	**mg/kg**	0.049	0.044	0.108	0.086	0.082	0.067	0.024	0.019
**Te**	**µg/kg**	< LOD*							
**V**	**µg/kg**	0.21	0.27	1.15	1.39	0.76	0.57	0.32	0.54
**Zn**	**mg/kg**	0.70	0.71	1.06	2.4	0.83	1.20	0.159	0.80

*LOD = 0.0001 µg/kg
